# Electronic‐State Polarization Engineering‐Regulated Fluorinated Covalent Organic Framework Nanocables for Fast Lithium‐Ion Storage

**DOI:** 10.1002/advs.75084

**Published:** 2026-03-29

**Authors:** Kaifu Xu, Jianfei Shi, Yuting Qin, Di He, Chengyin Wang, Tianyi Wang

**Affiliations:** ^1^ School of Chemistry & Materials Yangzhou University Yangzhou Jiangsu China; ^2^ Centre for Clean Energy Technology School of Mathematical and Physical Science Faculty of Science University of Technology Sydney Sydney New South Wales Australia

**Keywords:** electronic‐state polarization engineering, fluorinated covalent organic framework, high‐rate lithium‐ion storage, organic anode materials

## Abstract

Covalent organic frameworks (COFs) are promising anode materials for lithium‐ion batteries (LIBs) owing to their tunable porous structures and abundant redox‐active sites; however, insufficient electronic‐structure regulation and sluggish kinetics severely limit their rate capability and cycling stability. Herein, an electronic‐state polarization engineering strategy is proposed by in situ constructing fluorinated COF nanocables enriched with multipolar C─F units on conductive carbon nanotube scaffolds to form a high‐performance anode (F‐COF@CNT). The strong polarity of C─F bonds induces localized electronic polarization within the COF framework, enhancing electrostatic attraction toward Li^+^ and accelerating ion transport. Combined GITT analysis and density functional theory calculations reveal a reduced Li^+^ diffusion barrier and a sustained diffusion coefficient on the order of 10^−11^ cm^2^ s^−1^. Meanwhile, the interconnected CNTs network provides continuous electron‐transport pathways, effectively mitigating interfacial polarization and structural degradation. As a result, the F‐COF@CNT anode delivers 496.32 mAh g^−1^ at 0.1 A g^−1^ and maintains 281.21 mAh g^−1^ after 2000 cycles at 2.0 A g^−1^. An NCM811||F‐COF@CNT full cell retains 105.94 mAh g^−1^ after 500 cycles, demonstrating excellent practical stability. This work establishes electronic‐state polarization engineering as a general strategy to overcome kinetic limitations in organic framework anodes.

## Graphical abstract

Electronic‐state polarization engineering is realized by constructing fluorinated COF nanocables on conductive CNTs scaffolds. The polar C─F units generate localized electric fields and strong ion‐dipole interactions, promoting Li^+^ adsorption and transport. Together with the CNTs conductive network, the system enables fast ion‐electron kinetics and highly stable lithium storage.

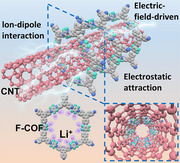

## 1 Introduction

Lithium‐ion batteries (LIBs) have become indispensable in modern energy storage due to their high energy density, long cycle life, and environmental benignity [1, 2, 3, 4, 5], yet conventional graphite anodes suffer from low theoretical capacity (372 mAh g^−1^) and limited rate capability, falling short of the demands of next‐generation high‐power applications [6]. Consequently, the design of advanced anode materials that combine high capacity, fast kinetics, and long‐term stability remains a central challenge in LIBs research [7, 8, 9, 10]. Covalent organic frameworks (COFs) have emerged as promising anode candidates owing to their highly ordered crystalline architectures [11, 12, 13], tunable porous networks, and abundant redox‐active sites [14, 15]. Their *π*–*π* conjugated backbones facilitate electron transport, and multiple organic functional groups offer potential sites for reversible Li^+^ insertion [16]. However, pristine COFs typically exhibit low intrinsic conductivity and poor reaction kinetics under high‐rate or extended‐cycling conditions, leading to increased polarization and structural degradation, thereby severely limiting practical performance [17, 18, 19, 20, 21].

Integrating COFs with conductive carbonaceous materials has been widely explored to address these limitations [22, 23, 24]. For example, in situ growth of COFs on carbon nanotubes (CNTs) has been shown to markedly improve capacity and cycle life in LIBs anodes by enhancing charge transport pathways and structural stability compared to purely physically mixed composites [25, 26]. Despite these advances, conventional approaches often rely on structural or morphological optimization alone, without direct modulation of the electronic structure governing the Li^+^ adsorption and transport kinetics [27, 28]. Beyond structural design [29], molecular polarity regulation has emerged as a highly effective yet underexplored strategy to modulate the interfacial ion‐electron interactions and reaction kinetics of COF‐based electrodes [30]. Incorporation of highly electronegative elements [31, 32], such as fluorine, can significantly modulate the electronic distribution within the framework, inducing localized internal electric fields that strengthen electrostatic attraction and ion‐pair interactions with Li^+^, potentially lowering diffusion barriers and accelerating interfacial reaction kinetics [33, 34]. Nevertheless, a mechanistic understanding of how polar electronic structures in fluorinated COFs influence lithium storage behavior and synergize with conductive networks is lacking due to limited experimental and theoretical evidence.

Motivated by these considerations, this study proposes an electronic‐state polarization engineering strategy, wherein fluorinated covalent organic framework nanocables enriched with polar C─F functional units are in situ constructed on carbon nanotube scaffolds (F‐COF@CNT) to achieve fast ion transport and efficient electron conduction. Combining electrochemical kinetic analysis (GITT) and density functional theory (DFT) calculations with multi‐scale structural characterization, this study systematically elucidates how C─F induced electronic polarization enhances Li^+^ adsorption and diffusion kinetics while CNTs provide a robust electron transport network. The resulting composite not only delivers high pseudocapacitive contribution and fast reaction kinetics with excellent rate capability and cycling stability in half battery, but also demonstrates stable performance in NCM811 full battery. This work establishes electronic‐state polarization regulation as a general and effective strategy to overcome kinetic bottlenecks in COF‐based anodes and provides new molecular and interfacial design principles for high‐rate Li^+^ storage materials.

## 2 Result and Discussion

**FIGURE 1:**
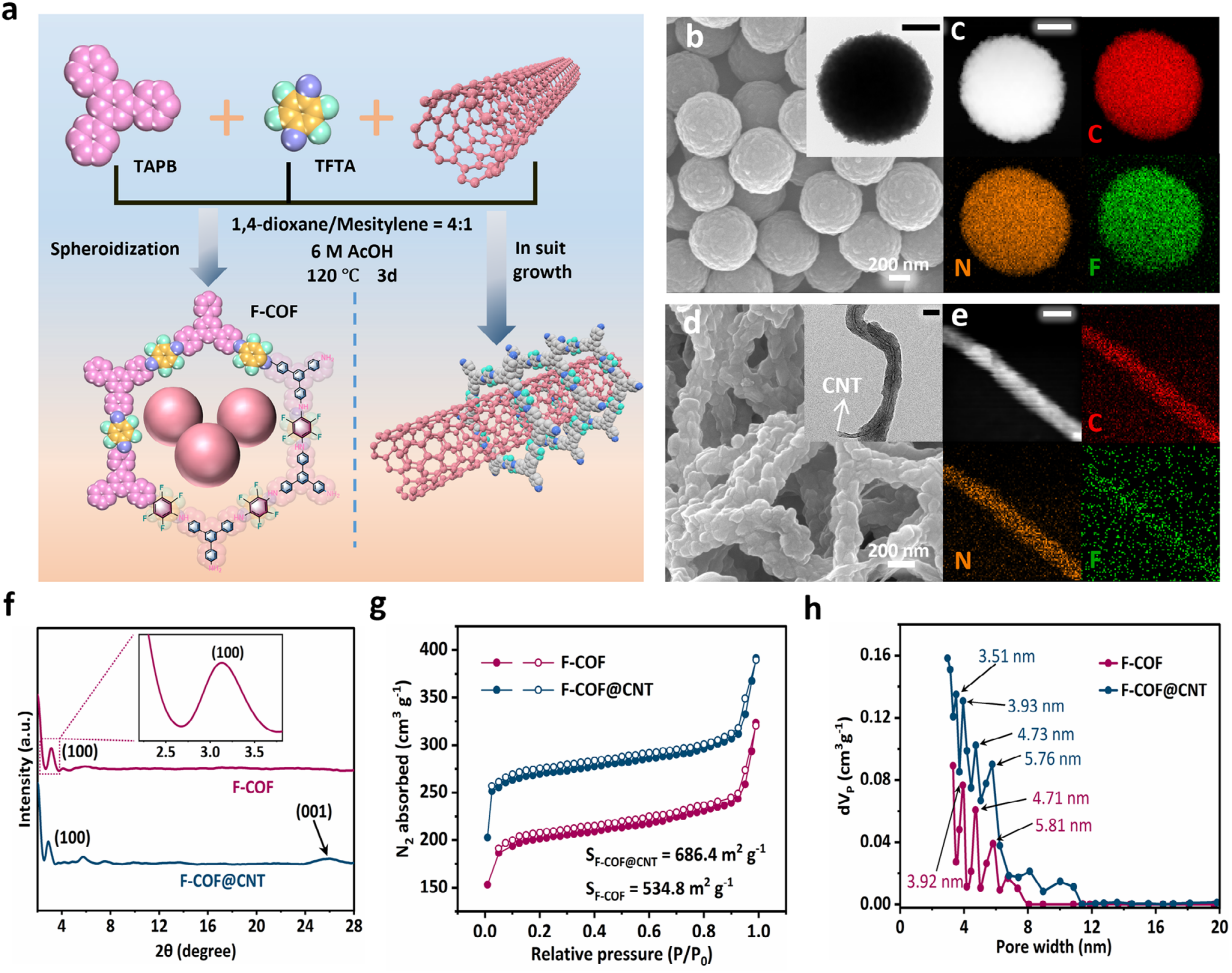
a) Schematic illustration of F‐COF spheres and F‐COF@CNT nanocables. b) SEM image of F‐COF spheres. The inset is the corresponding TEM image. c) The TEM image and the corresponding EDS mapping of F‐COF spheres. d) SEM image of F‐COF@CNT nanocables. The inset is the corresponding TEM image. e) The TEM image and the corresponding EDS mapping of F‐COF@CNT nanocables. f) XRD patterns of F‐COF spheres and F‐COF@CNT nanocables. g) N_2_ adsorption/desorption isotherms and h) the relevant pore size distributions of F‐COF spheres and F‐COF@CNT nanocables. The scale bars in the TEM images of (b–e) are 200 and 100 nm, respectively.

The F‐COF@CNT composite was prepared through a simple solvothermal method. First, CNTs were added to a mixed solvent consisting of 1,3,5‐trimethylbenzene (mesitylene) and 1,4‐dioxane, followed by sonication for 5 min to achieve a uniform dispersion. Subsequently, TAPB, TFTA, and appropriate aqueous acetic acid solution were added to the above dispersion, and the mixture was stirred at 300 rpm for 20 min. The resulting suspension was then transferred to a Schlenk tube and degassed through three freeze‐pump‐thaw cycles. The Schlenk tube was sealed under vacuum and heated at 120°C for 3 days. Finally, the product was dried overnight under vacuum at 80°C, affording the black TAPB‐TFTA‐COF@CNT material (denoted as F‐COF@CNT), as illustrated in the schematic procedure shown in Figure 1a. F‐COF can be obtained through the same process, except without CNTs. The macroscopic morphologies of F‐COF and F‐COF@CNT were examined by scanning electron microscopy (SEM). As shown in Figure 1b and Figure S1b, the synthesized COF sample exhibits highly uniform quasi‐spherical nanoparticle morphology with an average particle size of approximately 600 nm. The surface is covered with dense and continuous petal‐like protruding nano wrinkles. This hierarchical texturing of the spherical particles significantly increases the specific surface area and effective pore exposure, thereby providing shorter diffusion pathways and higher densities of active sites during ion or molecular transport processes. Furthermore, the protruded nanostructures on the surface enhance physical interparticle crosslinking and interfacial contact, offering a structurally stable and tunable interfacial foundation for subsequent compositing with CNTs. The high‐resolution transmission electron microscopy (HR‐TEM) image of the COF reveals a regular spherical morphology with clear overall particle contours. The interior of the particles displays distinct dark contrast regions, indicating a relatively dense internal structure. These morphological features indicate that F‐COF achieved uniform nucleation and growth, resulting in a structurally intact and ordered covalent organic framework. Benefiting from the *π*–*π* interactions between the 2D planes of CNTs and F‐COF, the SEM image in Figure 1d and Figure S1a and the HR‐TEM image in Figure 1d reveal that CNTs serve as the core skeleton, with their outer walls uniformly and completely coated by a COF layer. Notably, as shown in Figure 1c,e the corresponding elemental mapping (EDS) demonstrates homogeneous and spatially overlapped distributions of C, N, and F elements along the CNTs framework, directly confirming the conformal coating and in situ growth of fluorinated COF on CNTs rather than physical aggregation.

This coating layer is intimately interfaced with the carbon nanotubes, demonstrating successful in situ growth of COF on the CNTs surface and firm adhesion via *π*–*π* interactions. Such a composite structure not only effectively prevents agglomeration of COF nanoparticles but also establishes continuous conductive pathways, thereby contributing to enhanced electron transport efficiency and overall structural stability of the composite material. To gain deeper insights into the structure of the product, powder X‐ray diffraction (PXRD) analysis was performed. As shown in Figure 1f, pristine F‐COF exhibits characteristic diffraction peaks typical of a 2D covalent organic framework. The diffraction peak located at 2*θ* = 3.1° can be indexed to the (100) plane, indicating a periodically ordered framework structure [30], representing the periodic ordered arrangement within the framework plane. This diffraction pattern is highly consistent with the imine‐lined stacked COF model generated from the condensation of TAPB and TFTA, confirming the successful construction of a 2D organic framework with high crystallinity. After forming the F‐COF@CNT composite structure, the diffraction peak corresponding to the (100) plane remained clearly visible, indicating that the COF retained its intrinsic in‐plane ordered framework structure following in situ growth on the carbon nanotube surface. However, the peak intensity was slightly reduced and the peak shape was somewhat broadened compared to that of pristine F‐COF, reflecting a minor decrease in the crystallinity of the COF within the composite system. Such peak broadening is typically attributed to the reduced crystallite size and slight perturbation in interlayer stacking order resulting from the confined growth of COF on the CNT surface. Meanwhile, a broad shoulder peak appeared at 2*θ* = 25.8° in the high‐angle region, corresponding to the (001) plane, which arises from the interaction between the (002) plane of CNTs and the conjugated structure of the 2D F‐COF.

In the Small Angle X‐ray Scattering (SAXS) images shown in Figure S3, F‐COF exhibits intense and large‐sized scattering rings, confirming its highly uniform and regular spherical mesoscopic structure. In contrast, the scattering spots of F‐COF@CNT are contracted and weakened, indicating a transition of the COF framework from the original bulk periodic structure to a thin‐layer structure coated on the CNTs surface. The N_2_ adsorption‐desorption isotherms of F‐COF and F‐COF@CNT are presented in Figure 1g, both displaying type IV characteristics. The specific surface areas, calculated using the Brunauer–Emmett–Teller (BET) method, were 534.8 m^2^ g^−1^ for F‐COF and 686.4 m^2^ g^−1^ for F‐COF@CNT. Figure 1h compares the pore size distributions of F‐COF and F‐COF@CNT. Both materials are mainly mesoporous, however, the composite supported on CNTs shows more prominent pore volume peaks in the small‐to‐medium mesopore size range. The primary peaks for pristine COF are located at 3.92, 4.71, and 5.81 nm, while those for F‐COF@CNT shift toward a slightly narrower distribution, centered at 3.51, 3.93, 4.73, and 5.76 nm, with significantly enhanced peak intensities. This suggests that the composite material not only preserves the intrinsic mesoporous structure but also introduces additional accessible small‐to‐medium mesopores in the 3–6 nm range, resulting in an overall shift of the pore volume distribution toward higher values. Furthermore, the interconnected mesoporous network facilitates the maintenance of ion pathways within the electrode, mitigates the disconnection of active sites caused by structural pulverization during cycling, and thereby promotes charge transfer kinetics and enhances cycling stability.

**FIGURE 2:**
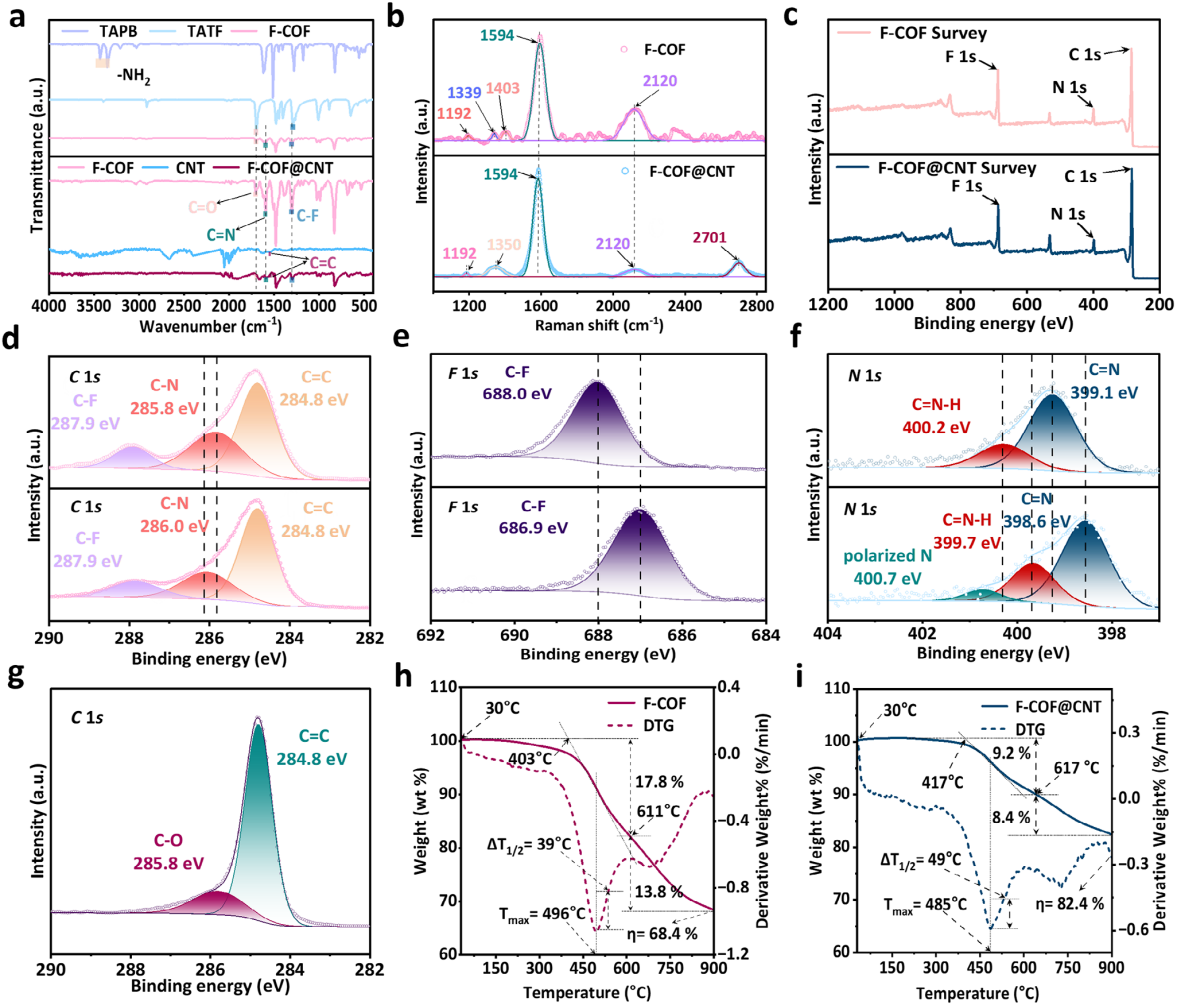
a) FTIR spectra of TAPB, TFTA, F‐COF spheres, CNT, and F‐COF@CNT nanocables. b) Raman spectra of F‐COF spheres and F‐COF@CNT nanocables. c) XPS survey spectra of F‐COF and F‐COF@CNT. d–f) High‐resolution C 1*s*, F 1*s*, N 1*s*, XPS spectra of F‐COF spheres and F‐COF@CNT nanocables. g) High‐resolution C 1*s* XPS spectra of CNT. h,i) TGA and DTG curves of F‐COF and F‐COF@CNT.

To further elucidate the structural composition, Fourier‐transform infrared spectroscopy (FT‐IR) and Raman spectroscopy X‐ray photoelectron spectroscopy measurements (XPS) analyses were performed. As shown in Figure 2a, the disappearance of the characteristic –NH_2_ stretching vibrations of TAPB at 3353 and 3430 cm^−1^ in the F‐COF spectrum confirms the successful condensation reaction [35]. The absorption peaks at 1299 cm^−1^, 1594 cm^−1^, and 1703 cm^−1^ are assigned to C─F, C═N, and C═O vibrations [36], respectively, while the emergence of the characteristic C═C vibration of CNTs at 1548 cm^−1^ in F‐COF@CNT evidences the successful integration of COF with CNTs. Raman spectra (Figure 2b) further reveal the structural evolution upon composite formation. The peaks at 1192, 1339, and 1403 cm^−1^ correspond to C─F stretching, aromatic C─C, and C─N vibrations, respectively, while the peak at 1594 cm^−1^ originates from C═N bonds, and the characteristic peak at 2120 cm^−1^ is attributed to the stretching vibration of C≡C. Upon CNTs incorporation, the two COF‐related peaks at 1339 and 1403 cm^−1^ merge into a broad band centered at 1350 cm^−1^, primarily due to the strong and broad D band of CNTs overlapping this region, together with interfacial coupling‐induced disorder and vibrational mode broadening. In addition, the characteristic 2D band of CNTs at 2701 cm^−1^ further confirms the successful construction of F‐COF@CNT nanocables [37].

As shown in Figure 2c–g, XPS measurements were employed to further investigate the elemental composition and chemical bonding states of the materials. Survey spectra show distinct C 1*s*, N 1*s*, and F 1*s* signals for both samples, verifying the formation of fluorinated COF. The enhanced C 1*s* intensity and relatively weaker N and F signals in F‐COF@CNT indicate a uniform COF coating on the CNTs surface, consistent with electron microscopy observations. The high‐resolution C 1*s* spectrum of F‐COF can be deconvoluted into three characteristic components located at 284.8, 285.8, and 287.9 eV, which are assigned to C═C, C─N, and C─F bonds, respectively [38, 39]. Reflecting the conjugated framework, imine linkages, and fluorinated units within the COF structure. After composite formation, the C─N peak exhibits a slight positive shift to 286.0 eV, whereas the C─F peak shifts toward lower binding energy to approximately 287.0 eV, suggesting pronounced electronic coupling between the COF and CNTs. The highly electronegative fluorine atoms exert strong electron‐withdrawing effects within the COF framework, thereby inducing charge redistribution and localized electronic polarization. The presence of C─F bonds enhances the electron‐withdrawing capability and surface polarity of the material, which is beneficial for improving the chemical stability of the COF as well as the electrochemical interfacial properties [40]. Correspondingly, the F 1s peak shifts from 688.0 eV in pristine F‐COF to 686.9 eV in F‐COF@CNT (Figure 2e), indicating increased electron density on F atoms due to interfacial charge transfer. Notably, as shown in Figure 2f, the high‐resolution N 1*s* spectra provide direct evidence of electronic‐state polarization. Pristine F‐COF exhibits two components at 399.1 eV (C═N) and 400.2 eV (C═N─H). In contrast, the dominant imine N peak in F‐COF@CNT shifts to a lower binding energy (∼398.6 eV), indicating electron enrichment at the nitrogen sites. Meanwhile, a new component appears at a higher binding energy (∼400.7 eV), which is attributed to electron‐deficient nitrogen species induced by polarization‐driven charge redistribution between the fluorinated COF framework and the CNTs conductive network. The coexistence of electron‐enriched and electron‐deficient nitrogen environments suggests the formation of localized electronic polarization within the COF framework. Such polarization can strengthen electrostatic interactions with Li^+^ and facilitate Li^+^ adsorption and transport along the framework channels. To further verify the interfacial interaction characteristics, the C 1*s* spectra of pristine CNTs and F‐COF@CNT were compared. The relative intensity of the C═C signal in the F‐COF@CNT composite is noticeably attenuated, which can be attributed to a surface coverage effect induced by the in situ growth of the COF on CNTs. This coating partially shields the sp^2^ carbon signals originating from the CNTs substrate, thereby reducing their apparent contribution in the photoelectron spectrum [41].

Figure 2h,i presents the thermogravimetric analysis (TGA) and derivative thermogravimetric (DTG) curves of F‐COF and F‐COF@CNT measured under a nitrogen atmosphere. For both materials, the slight mass loss observed in the temperature range of 30°C–400°C can be primarily attributed to the removal of adsorbed moisture and residual solvents, indicating good structural stability at ambient temperature. In contrast, pronounced differences are observed in the main thermal decomposition region. For F‐COF, the primary weight‐loss region is located between 403°C and 611°C, with a maximum decomposition rate temperature (T_max_) of 496°C and a total weight loss of approximately 31.6%, of which about 17.8% occurs in the main decomposition stage, leaving a residual mass fraction (η) of 68.4%. Upon CNTs incorporation, the main decomposition region of F‐COF@CNT shifts slightly to a higher temperature range of 417°C–617°C, with a T_max_ of 485°C. Notably, the total weight loss is reduced to approximately 17.6%, with only 9.2% occurring in the principal decomposition stage, the residual mass fraction significantly increases to 82.4%. In addition, the half‐peak width (ΔT_1/2_) increases from 39°C to 49°C, indicating a more gradual and broadened decomposition process for F‐COF@CNT. Collectively, these comparative results demonstrate that the incorporation of CNTs effectively enhances the thermal stability of the composite. Such improvement can be attributed to the synergistic interplay of multiple physical and chemical stabilization effects. From a thermal transport perspective, CNTs possess exceptionally high thermal conductivity and intrinsic thermal stability. Their incorporation into the composite establishes a continuous heat‐conduction network that efficiently dissipates thermal flux and mitigates localized overheating, thereby suppressing the rapid degradation of the COF framework. In addition, the 3D network of CNTs provides robust physical reinforcement for the COF, constraining structural collapse and the release of volatile fragments during thermal decomposition, which renders the degradation process more gradual. More importantly, pronounced *π*–*π* interactions and van der Waals forces exist at the CNTs/COF interface, facilitating interfacial chemical crosslinking and carbonization processes. Such interfacial effects can induce the rearrangement and enhanced conjugation of aromatic domains at elevated temperatures, leading to the formation of a denser and more stable carbonaceous residue layer. As a result, incorporating CNTs enables the COF‐based material to retain structural integrity and electrical continuity at high temperatures, highlighting its substantial potential as a conductive scaffold for electrode applications.

**FIGURE 3:**
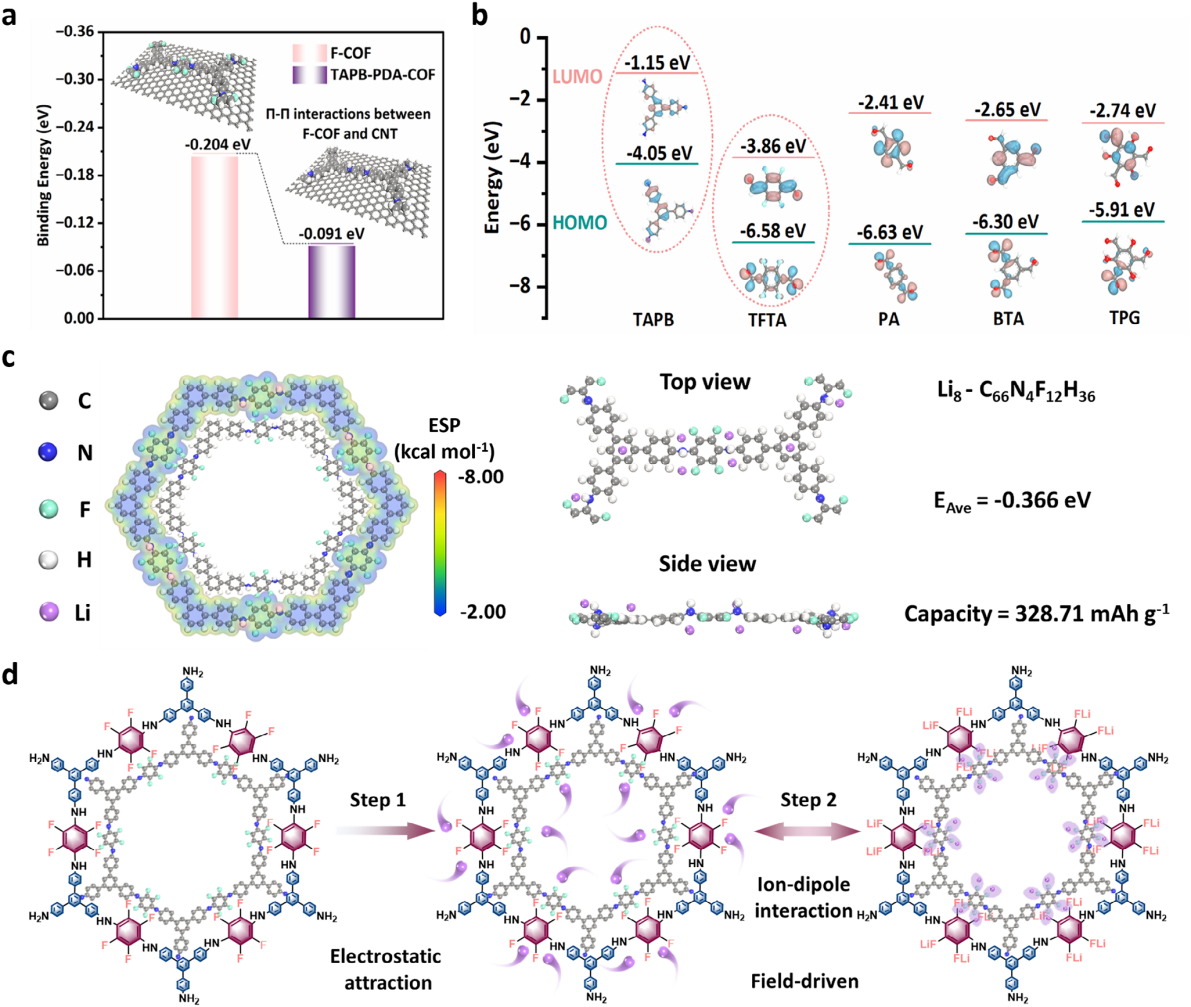
a) Comparison of the binding energies between TAPB‐TFTA‐COF (F‐COF), TAPB‐PDA‐COF, and CNT. b) HOMO/LUMO energy levels and orbital distributions of representative COF monomers. c) Electrostatic potential distribution and optimized Li‐binding configurations of F‐COF. d) Structure evolution of F‐COF during discharge/charge.

Density functional theory (DFT) calculations were performed to gain deeper insight into the interfacial interactions between F‐COF and CNTs, as illustrated in Figure 3a. After geometric optimization, the adsorption energy of F‐COF on the CNTs surface was calculated to be −0.204 eV, indicating the formation of a stable adsorption configuration. In contrast, the TAPB‐PDA‐COF derived from 1,3,5‐tris(4‐aminophenyl) benzene and terephthalaldehyde exhibits a much weaker adsorption energy of only −0.091 eV on the CNTs surface. Since a more negative adsorption energy corresponds to stronger interfacial interaction, these results suggest that F‐COF possesses a significantly higher affinity toward CNTs and is therefore more likely to undergo ordered growth on the CNTs surface. This enhanced interaction can be attributed to the fluorinated structure of the TFTA units, which markedly strengthens the interfacial affinity between the COF and CNTs. In addition, the high electronegativity of fluorine induces stronger dipole moments and polarization effects, thereby promoting more stable adsorption of the molecular fragments on the CNTs surface.

To further clarify the structural rationale for selecting TAPB and TFTA for high‐performance COF‐based electrodes, frontier molecular orbital calculations were performed for several conventional COF building blocks (PA, BTA, and TPG). As shown in Figure 3b, TFTA exhibits the lowest LUMO energy level together with highly polar C─F moieties, rendering it an excellent electron acceptor and an effective Li^+^ adsorption center. Meanwhile, TAPB possesses the highest HOMO energy level and a highly delocalized π‐conjugated structure, making it an efficient electron donor. The combination of TAPB and TFTA thus forms a strong donor‐acceptor (D‐A) coupled system, which significantly enhances the polarization capability, electron transport efficiency, and Li^+^ anchoring activity of the resulting COF—advantages that are not achievable with other monomers such as PA, BTA, or TPG. Consequently, the TAPB/TFTA pairing constructs an energetically optimal framework for lithium storage reactions at the molecular level.

As shown in Figure 3c, DFT optimization of the Li_8_‐C_66_N_4_F_12_H_36_ configuration reveals that eight Li^+^ can be stably anchored around the pore walls of the COF. The calculated average adsorption energy is −0.366 eV, which falls within the moderate charge‐controlled adsorption regime, indicating favorable and reversible Li^+^ binding behavior. Such a magnitude of adsorption energy thermodynamically guarantees stable Li^+^ insertion while avoiding excessively strong ionic bonding or covalent character, thereby providing a favorable reversibility window for subsequent Li^+^ extraction. Further electrostatic potential (ESP) analysis reveals that the fluorine atoms in the TFTA units constitute the most negatively charged regions within the framework, exhibiting markedly lower local electrostatic potentials than the surrounding backbone. These highly polarized sites act as dominant electron‐accepting centers, providing energetically favorable anchoring sites for Li^+^ adsorption. Notably, the Li atoms are uniformly and non‐aggregately distributed along the pore walls, indicating that the system effectively suppresses Li clustering. This feature is critical for preventing localized metallization, potential drift, and undesirable lithium plating, and is therefore essential for achieving long‐term cycling stability. Moreover, the calculated theoretical capacity of the F‐COF reaches 328.71 mAh g^−1^, which is in excellent agreement with the experimentally measured value of 325.27 mAh g^−1^, further validating the proposed lithium storage mechanism. It should be noted that this theoretical capacity is derived from the optimized Li_8_‐C_66_N_4_F_12_H_36_ adsorption model and mainly reflects the intrinsic Li‐storage contribution from the dominant anchoring sites within the fluorinated COF framework. In contrast, the higher experimental capacity of the F‐COF@CNT electrode at low current density cannot be fully explained by the simplified theoretical model. It mainly arises from additional storage contributions, including secondary redox‐active sites, interfacial pseudocapacitive storage at the F‐COF/CNT heterointerface, and charge storage enabled by the hierarchical porous architecture. As illustrated in Figure 3d, the structural evolution during electrochemical cycling can be described as follows: during the charging process, Li^+^ is electrostatically attracted to the highly electronegative F atoms, forming Li─F electrostatic interaction; upon discharging, the applied external electric field drives the reversible extraction of Li^+^ from these binding sites.

**FIGURE 4:**
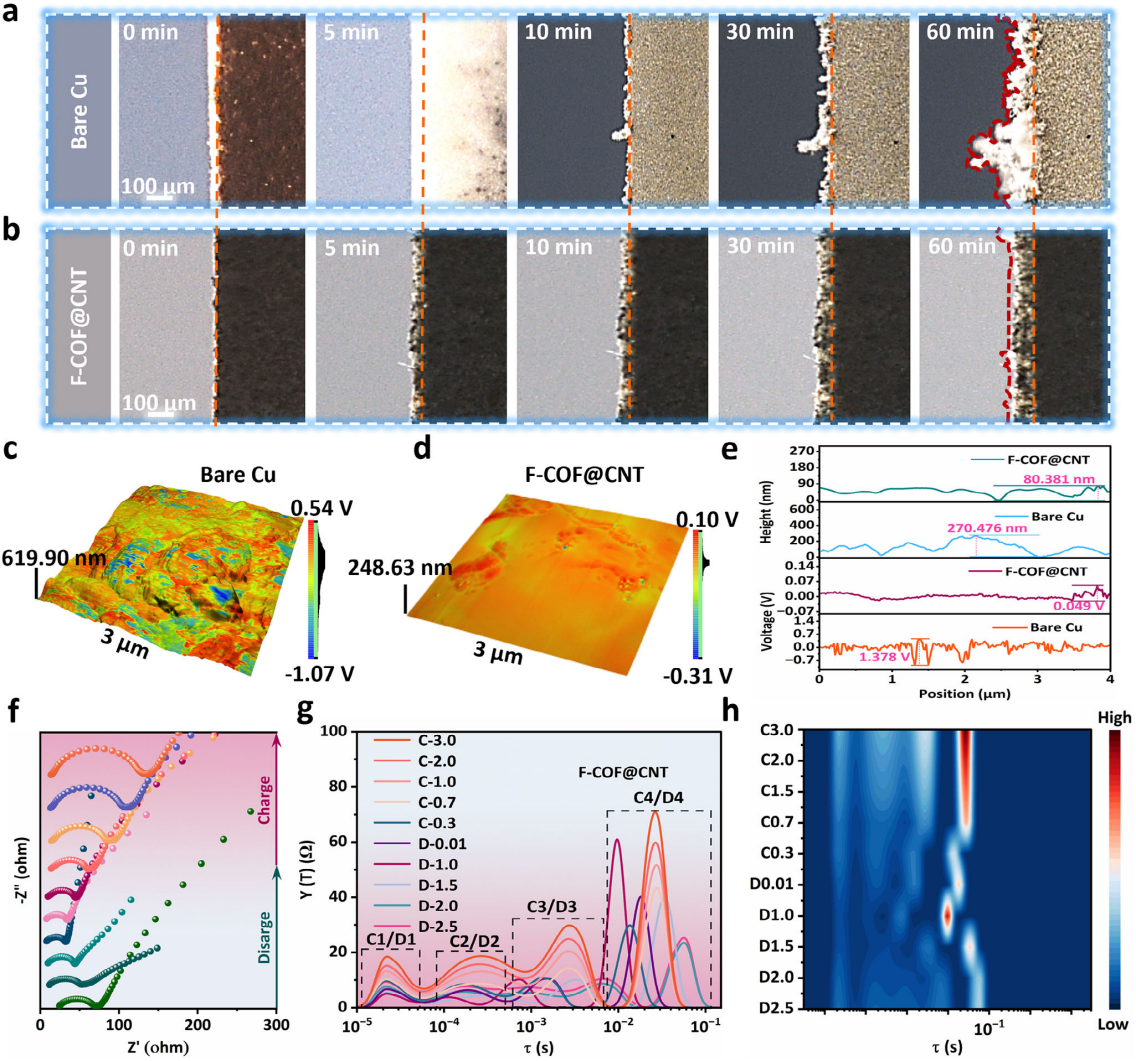
a,b) In situ optical images of Li deposition on bare Cu and F‐COF@CNT. The current density and areal capacity were 1 mA cm^−2^ and 1 mAh cm^−2^, respectively. c,d) Morphology of the lithium deposition layers and the corresponding potential distributions on bare Cu and F‐COF@CNT surfaces. e) Diagonal height fluctuation and potential fluctuation analysis of the height image and the potential difference image of the KPFM of the lithium deposited layer. f,g) In situ EIS Nyquist plots during charge/discharge and corresponding DRT spectra. h) DRT contour map of relaxation processes.

To further elucidate the role of F‐COF@CNT nanocables in regulating Li deposition behavior, in situ optical microscopy was employed to monitor Li plating on bare Cu and F‐COF@CNT electrodes (Figure 4a). On bare Cu, Li deposition exhibits pronounced nucleation heterogeneity from the initial stage. Within 5–10 min, numerous bright deposition sites emerge and rapidly evolve into dendritic protrusions, which further develop into large‐area radial dendrites upon prolonged deposition (30–60 min), indicating severe nucleation inhomogeneity and kinetically uncontrolled dendritic Li deposition behavior. In contrast, the F‐COF@CNT electrode maintains a uniform and continuous Li deposition morphology throughout the entire plating process (Figure 4b). Even after 60 min, a dense and smooth Li layer is preserved without observable dendritic features, demonstrating effective interfacial regulation and deposition stability. Kelvin probe force microscopy (KPFM) analysis (Figure 4c–e) further reveals that the Li deposition layer on bare Cu is highly rough and inhomogeneous, with height fluctuations up to ∼619.9 nm and a broad surface potential distribution (−1.07 to 0.54 V), indicative of severe local current concentration and electric field distortion. By comparison, the Li layer formed on COF@CNT exhibits markedly reduced height fluctuation (∼248.6 nm) and a significantly narrowed potential distribution (−0.31 to 0.10 V), reflecting a homogenized interfacial electric field and stabilized Li deposition behavior. Diagonal line profile analysis further reveals that the F‐COF@CNT electrode exhibits a markedly reduced height fluctuation (∼80.4 nm) and surface potential fluctuation (∼0.049 V), both of which are substantially lower than those of bare Cu (270.5 nm and 1.378 V, respectively). This concurrent homogenization of surface morphology and potential distribution indicates that the F‐COF@CNT interface effectively regulates the local electric field during Li deposition, thereby promoting uniform nucleation and suppressing unstable growth. Meanwhile, in situ electrochemical impedance spectroscopy (EIS) was carried out to probe the interfacial kinetic evolution during Li deposition (Figure 4f–h). The Nyquist plots show a reversible decrease in charge‐transfer resistance during lithiation, while the corresponding DRT spectra resolve the relaxation processes associated with interfacial charge transfer and Li^+^ diffusion. The DRT contour map further confirms stable and low‐polarization interfacial kinetics throughout the deposition process. These results collectively demonstrate that the F‐COF@CNT interface effectively regulates the local electric field and charge‐transfer behavior during Li deposition, arising from the synergistic effects of polar C─F functional units that induce localized Li^+^ attraction and the continuous CNTs network that ensures uniform electron transport.

**FIGURE 5:**
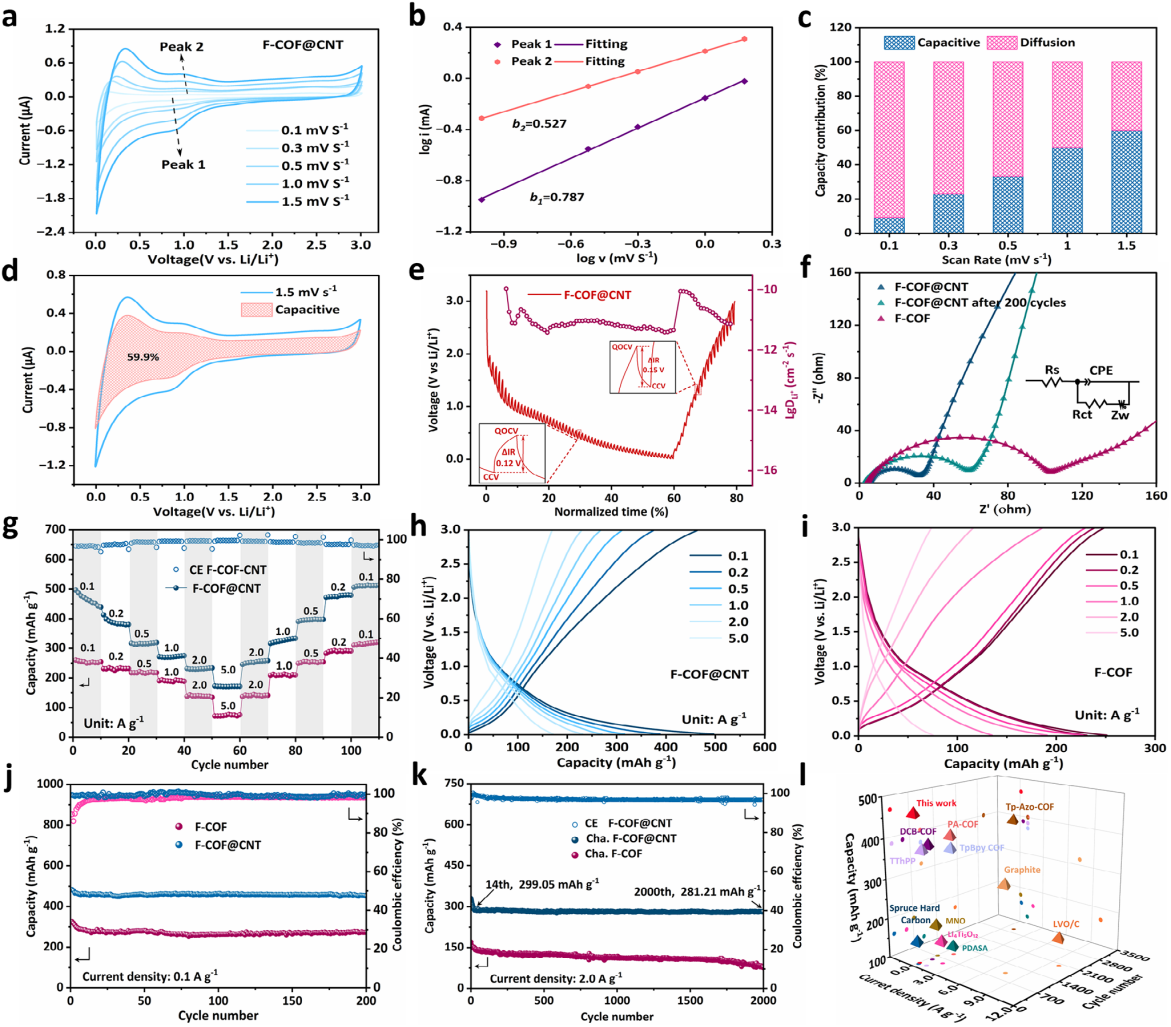
a) CV curves from 0.1 to 1.5 mV s^−1^, b) fitting lines of log (peak current) vs log (scan rate), and c) Capacitive contribution ratio at diverse sweep rates of F‐COF@CNT nanocables. d) Capacitive contribution of F‐COF@CNT nanocables at 1.5 mV s^−1^. e) GITT diagram and Li^+^ diffusion coefficient of F‐COF@CNT nanocables. f) Nyquist plots of F‐COF spheres, F‐COF@CNT nanocables, and F‐COF@CNT nanocables after 200 cycles. g) Rate performance and h,i) discharge/charge profiles at various current densities of F‐COF spheres and F‐COF@CNT nanocables. j) Cycling performance of F‐COF spheres and F‐COF@CNT nanocables. k) Long‐term cycling performance of F‐COF spheres and F‐COF@CNT nanocables. l) Comparison of Li storage properties of F‐COF@CNT nanocables with other anode materials.

To investigate their electrochemical properties, F‐COF microspheres and F‐COF@CNT nanocables were fabricated as anodes for LIBs and evaluated within a voltage window of 0.01–3.0 V vs Li^+^/Li. As shown in Figure 5a and Figure S7, cyclic voltammetry (CV) curves of both electrodes recorded at scan rates from 0.1 to 1.5 mV s^−1^ exhibit well‐defined and highly reversible redox features with negligible peak distortion, indicating good structural stability and electrochemical reversibility. The gradually increased peak currents with scan rate suggest a combination of diffusion‐controlled and surface capacitive Li^+^ storage processes. The charge transfer behavior was analyzed according to Equations (1) and (2):

(1) $$i=av^{b}$$

(2) logi=loga+blogv

Here, α is a constant, *i* and *v* represent the peak current and scan rate, respectively, and the Li^+^ storage kinetics can be evaluated by the *b* value. In general, the *b* value close to 0.5 indicates a diffusion‐controlled process, whereas a value approaching 1.0 corresponds to a capacitive‐controlled process. As shown in Figure 5b, the extracted b values of 0.527 (Peak 1) and 0.787 (Peak 2) indicate mixed diffusion‐capacitive behavior, with a pronounced capacitive contribution. Notably, the relatively high *b* values suggest a pronounced surface capacitive contribution, which can be attributed to the abundant electrochemically active sites provided by the porous COF framework and the highly conductive network constructed by CNTs, together facilitating rapid electron and ion transport. This synergistic effect accounts for the superior Li^+^ storage kinetics and high reversible capacity of the F‐COF@CNT nanocables. To further quantify the capacitive contribution, the current response was analyzed according to Equation (3):

(3) $$i\;\left(V\right)=k_{1}\;v+k_{2}v^{1/2}$$

Here, *k_1_* and *k_2_* are constants, where *k_1_v* represents the capacitive‐controlled contribution and *k_2_v* ^1/2^ corresponds to the diffusion‐controlled contribution. The relative contributions of capacitive and diffusion‐controlled processes at different scan rates are summarized in Figure 5c. With increasing scan rate, the proportion of the capacitive contribution gradually increases, indicating efficient Li^+^ storage and rapid electrochemical response. At a scan rate of 1.5 mV s^−1^, the pseudocapacitive contribution reaches 59.9%, as shown in Figure 5d. The high capacitive contribution is attributed to the synergistic integration of the polar, porous COF framework and the continuous CNTs conductive network, which provides abundant surface‐active sites and rapid electron/ion transport pathways.

This pronounced capacitive contribution is attributed to the unique hierarchical conductive and porous architecture of the F‐COF@CNT system. The CNTs provide continuous electron‐conduction pathways, while the C─N and C─F functional sites within the COF framework not only serve as reversible lithiation centers but also induce localized electronic structure modulation, thereby enhancing interfacial electron transfer and Li^+^ adsorption kinetics [43]. Fluorination enhances interfacial polarity and lithiophilicity, promoting synergistic electron and Li^+^ transport. To gain deeper insight into the intrinsic mechanisms underlying the superior electrochemical performance of the F‐COF@CNT electrode, its Li^+^ diffusion kinetics and interfacial charge‐transfer behavior were systematically investigated using the galvanostatic intermittent titration technique (GITT) and EIS, respectively. As shown in Figure 5e, the GITT profiles display small and reversible voltage steps with low polarization (∼0.12–0.15 V), indicating fast Li^+^ transport. The calculated Li^+^ diffusion coefficients remain at a relatively high level of ∼10^−11^ cm^2^ s^−1^ throughout the lithiation/delithiation process. This behavior originates from the porous COF framework that provides abundant Li^+^ diffusion pathways, while the CNTs conductive network enables rapid electron transport. In addition, fluorination effectively enhances the polarity and lithiophilicity of the COF backbone, inducing interfacial charge redistribution and further lowering the Li^+^ diffusion energy barrier, thereby promoting fast ion insertion and extraction. EIS measurements further corroborate the pronounced advantages of the composite electrode in terms of coupled electron/ion transport [44]. As shown in Figure 5f, F‐COF@CNT exhibits a charge‐transfer resistance (R_ct_ = 34 Ω), which is significantly lower than that of the pristine F‐COF electrode (101 Ω), and shows only a slight increase after prolonged cycling, demonstrating stable interfacial kinetics. This result is in good agreement with the cyclic voltammetry and kinetic analyses discussed above, further confirming the existence of strong interfacial coupling effects and fast charge‐transfer behavior in the composite electrode [45]. Rate capability tests (Figure 5g–i) show that the F‐COF@CNT electrode delivers high reversible capacities of 496.32, 387.81, 315.77, 270.13, and 230.94 mAh g^−1^ at current densities from 0.1 to 5.0 A g^−1^, consistently outperforming pristine F‐COF. Notably, when the current density is switched back to 0.1 A g^−1^, both the F‐COF and F‐COF@CNT electrodes not only fully recover their original capacities but also exhibit an obvious capacity increase. This behavior suggests a typical electrochemical activation process during cycling, associated with improved ion/electron transport and gradual activation of electrochemically accessible sites.

High‐rate cycling promotes electrolyte penetration into the porous COF framework, activating internal pores and previously inaccessible redox‐active sites. In F‐COF@CNT, the mesoporous COF framework and 3D CNTs scaffold enable deeper electrolyte penetration and improved Li^+^ accessibility, thus enhancing Li^+^ accessibility and reaction activity within the porous framework. Meanwhile, repeated cycling improves interfacial contact and strengthens *π*–*π* interactions between COF and CNTs, resulting in reduced interfacial resistance and improved electrical connectivity.

More importantly, the continuous CNTs network provides rapid electron transport, while the ordered COF channels facilitate fast Li^+^ diffusion, thereby suppressing both charge‐transfer and concentration polarization during cycling. In addition, the C─F‐induced electronic polarization creates localized electrostatic interaction sites for Li^+^, promoting uniform Li^+^ adsorption and transport within the framework. The synergistic regulation of electron conduction, ion diffusion, and polarization‐induced Li^+^ distribution effectively mitigates polarization buildup and stabilizes the electrode/electrolyte interface during prolonged cycling. As a result, the electrode exhibits higher reversible capacity and reduced voltage polarization. As shown in Figure 5j–k, the long‐term cycling performance further confirms the outstanding durability of the composite electrode. The F‐COF@CNT electrode retains 98% of its initial capacity after 200 cycles, with the Coulombic efficiency remaining above 97.5%. Even at a high current density of 2.0 A g^−1^, the F‐COF@CNT electrode maintains a reversible capacity of 281.21 mAh g^−1^ after 2000 cycles, corresponding to a capacity retention of 94%. In sharp contrast, the pristine F‐COF electrode suffers from pronounced capacity decay under identical conditions. Notably, the high capacity of the F‐COF@CNT electrode at low current densities ranks among the best reported for organic anode materials and also demonstrates competitive advantages over many other anode systems, as summarized in Figure 5l and Table S1 [41, 42, 43, 44, 45, 46, 47, 48, 49, 50, 51]. Given the excellent Li^+^ storage capability of the F‐COF@CNT nanocables, a full battery was assembled using NCM811 as the cathode and F‐COF@CNT as the anode. As shown in Figures S9–S11, the NCM811||F‐COF@CNT full battery delivers a reversible capacity of 105.94 mAh g^−1^ after 500 cycles at 0.5 A g^−1^, demonstrating stable cycling performance under practical operating conditions.

## 3 Conclusion

This work presents an interface design strategy based on fluorine‐induced electronic‐state polarization engineering to enable fast and stable lithium storage. Fluorinated covalent organic framework nanocables were in situ constructed on a continuous carbon nanotube scaffold (F‐COF@CNT), achieving synergistic regulation of electronic structure and charge transport at both molecular and structural levels. The strongly polar C─F functional units generate localized electric fields that enhance electrostatic attraction and ion‐pair interactions toward Li^+^, as supported by DFT‐calculated moderate Li^+^ adsorption energies (−0.366 eV), enabling reversible and energetically favorable Li^+^ binding within the COF channels. Meanwhile, the CNTs conductive backbone establishes continuous electron‐transport pathways, effectively suppressing interfacial polarization and structural degradation, as evidenced by reduced nucleation overpotential and homogenized potential distribution during Li deposition. As a result, the F‐COF@CNT electrode delivers a high pseudocapacitive contribution (∼60%), excellent reversible capacity (496.32 mAh g^−1^ at 0.1 A g^−1^), and outstanding long‐term durability, retaining 281.21 mAh g^−1^ after 2000 cycles at 2.0 A g^−1^. Furthermore, the assembled NCM811||F‐COF@CNT full battery maintains 105.94 mAh g^−1^ after 500 cycles with low polarization, demonstrating robust device‐level stability. Overall, this work establishes electronic‐state polarization engineering as an effective strategy to reconcile high lithium affinity with rapid interfacial kinetics, providing a rational design paradigm for high‐rate and durable organic framework anodes.

## Funding

This work was supported by the National Natural Science Foundation of China (Grant No. 22472145) and Postgraduate Research & Practice Innovation Program of Jiangsu Province (Yangzhou University) (KYCX24_3729).

## Conflicts of Interest

The authors declare no conflicts of interest.

## Supporting information




**Supporting file**: advs75084‐sup‐0001‐SuppMat.docx

## Data Availability

The data that support the findings of this study are available in the supplementary material of this article.
