# Supplementary material for: Electronic‐State Polarization Engineering‐Regulated Fluorinated Covalent Organic Framework Nanocables for Fast Lithium‐Ion Storage
Source: Adv Sci (Weinh). 2026 Mar 29;13(34):e75084. doi: 10.1002/advs.75084 (PMC13285149; doi:10.1002/advs.75084)
Supplement: Supplementary file 1 — Supporting file: advs75084‐sup‐0001‐SuppMat.docx [file ADVS-13-e75084-s001.docx]

Supporting Information

Electronic-State Polarization Engineering-Regulated Fluorinated Covalent Organic Framework Nanocables for Fast Lithium-Ion Storage

*Kaifu Xu ^1^, Jianfei Shi ^1^, Yuting Qin ^1^, Di He ^1,^* **, Chengyin Wang ^1,^* **, Tianyi Wang^1, 2,^* *

^1^ School of Chemistry & Materials, Yangzhou University, 180 Si-Wang-Ting Road, Yangzhou, Jiangsu 225002, China

^2^ Centre for Clean Energy Technology, School of Mathematical and Physical Science, Faculty of Science, University of Technology Sydney, Sydney, NSW 2007, Australia.

E-mail: (D. He) [yzhedi@foxmail.com](mailto:yzhedi@foxmail.com); (C.Y. Wang) [wangcy@yzu.edu.cn](mailto:wangcy@yzu.edu.cn); (T.Y. Wang) [Tianyi.wang@uts.edu.au](mailto:Tianyi.wang@uts.edu.au)

**1. Experimental Section**

**1.1 Materials preparation**

All starting chemicals and reagents were purchased from commercial suppliers and used without further purification. 1,3,5-Tris(4-aminophenyl) benzene (TAPB, 98%), 2,3,5,6-tetrafluoroterephthalaldehyde (TFTA, 99%), acetic acid (99%), mesitylene (99%), ethanol (99%), tetrahydrofuran (99%), and N, N-dimethylformamide (99%), were purchased from Adamas (Shanghai, China). Carbon nanotubes (CNTs; diameter 10-20 nm, length 10-30 μm), 1,4-Dioxane (99%) were acquired from Macklin (Shanghai, China). The electrolyte was 1 M LiPF6 (ethylene carbonate (EC)/dimethyl carbonate (DMC)=1:1), acquired from Sigma-Aldrich (USA), 99.95%.

**1.2 Synthesis of F-COF**

The synthesis of TAPB-TFTA-COF was carried out as follows: TAPB (0.16 mmol, 56.2 mg) and TFTA (0.24 mmol, 49.5 mg) were added to a Schlenk tube containing a solvent mixture of 2 mL of 1,3,5-trimethylbenzene (mesitylene) and 8 mL of 1,4-dioxane. The mixture was then sonicated for 10 min to form a homogeneous suspension. Subsequently, 0.8 mL of a 6 mol L^-1^ aqueous acetic acid solution was added as the catalyst, followed by another 10 min of sonication. The tube containing the reaction mixture underwent three freeze-pump-thaw cycles for degassing, was sealed under vacuum, and heated to 120 °C for 3 days. The resulting precipitate was collected and washed thoroughly with mesitylene, 1,4-dioxane, and acetone, respectively. Finally, the product was dried under vacuum at 80 °C for 24 h, yielding a brownish-yellow powder (denoted as F-COF).

**1.2 Synthesis of F-COF@CNT**

The F-COF@CNT composite was prepared as follows: First, 27.8 mg of CNTs was added to a mixed solvent consisting of 2 mL of 1,3,5-trimethylbenzene (mesitylene) and 8 mL of 1,4-dioxane, followed by sonication for 5 min to achieve a uniform dispersion. Subsequently, TAPB (0.16 mmol, 56.2 mg), TFTA (0.24 mmol, 49.5 mg), and 0.8 mL of a 6 mol L^-1^ aqueous acetic acid solution were added to the above dispersion, and the mixture was stirred at 300 rpm for 20 min. The resulting suspension was then transferred to a Schlenk tube and degassed through three freeze-pump-thaw cycles. The Schlenk tube was sealed under vacuum and heated at 120 °C for 3 days. Finally, the product was dried overnight under vacuum at 80 °C, affording the black TAPB-TFTA-COF@CNT material (denoted as F-COF@CNT).

**1.3 Preparation of electrodes and assembly of batteries**

The COF active material, the conductive agent carbon black, and the binder polyvinylidene fluoride (PVDF) were dried in a vacuum oven at 60 °C for 12 h before use. The dried COFs material or its composite material (F-COF@CNT), the carbon black, and the PVDF were mixed in a mass ratio of 7: 2: 1, and ground clockwise in a mortar for 20 min. The mixed powder was added with an appropriate amount of anhydrous NMP reagent, and the homogeneous slurry was obtained after thorough mixing. The slurry was uniformly coated on the surface of the copper foil by a spatula, and was vacuum dried at 60 °C for 24 h to remove the N-Methyl pyrrolidone (NMP) solvent. Cut the dried electrode sheet into a 12 mm diameter round sheet, and weigh the mass for use. CR2032 coin battery was assembled in an argon-filled glove box (water < 0.1 ppm, oxygen < 0.1 ppm) using the prepared electrode as the anode, lithium metal sheet as the counter electrode, and 1 M LiPF_6_ (ethylene carbonate (EC): dimethyl carbonate (DMC)=1: 1) as the electrolyte, Celgard 2400 as separator. After the battery is assembled, it is left to stand for 24 h before the electrochemical performance test. All the specific capacities are determined based on the area mass of the active materials, and the mass loading of the active material on the working electrodes was controlled in the range of 0.6-1.0 mg cm^-2^, with an average loading of approximately 0.8 mg cm^-2^, and all electrochemical tests were conducted using electrodes with comparable loadings.

**2. Characterization**

Scanning electron microscopy (SEM) images were obtained by Zeiss-Supra 55 microscopes at an acceleration voltage of 5 kV. Transmission electron microscopy (TEM) and Energy Dispersive Spectrometer (EDS) elemental mapping scans were recorded using Tecnai G2 F30 S-TWIN at an acceleration voltage of 300 kV. The high-resolution Kelvin probe force microscopy (KPFM) is the SPM-9700HT from Shimadzu Corporation, Japan. Bruker AXS D8 Advance performed the Powder X-ray diffraction (PXRD) patterns with Cu Kα radiation of 40 kV (λ=1.5418 Å). Fourier transform infrared (FTIR) spectra were obtained with 670-IR+610-IR from Varian, USA. Top-mounted diffuse reflectance attachment (diamond attachment). The spectral range is 4000-400 cm^-1^. Thermogravimetric analysis (TGA) was carried out on a NETZSCH STA 449 F3 thermogravimetric analyzer. The N_2_ adsorption-desorption isothermals and pore size distribution were obtained by Autosorb-Iq via the Brunauer-Emmet-Teller (BET) method. XPS analysis was carried out using a Thermo Scientific ESCALAB 250Xi X-ray photoelectron spectrometer with Al Kα radiation of 1486.6 eV as the excitation source, the survey thickness is 2-3 nm. The reference for calibration is the peak of C 1*s* at 284.8 eV.

**3. Theoretical Calculations.**

All calculations were completed in the Materials Studio 2020. The DMol^3^ module was used to calculate the HOMO-LUMO and electrostatic potential (ESP). The geometries of the COF molecular fragments were fully optimized using density functional theory (DFT) with the generalized gradient approximation (GGA) and the Perdew-Burke-Ernzerhof (PBE) exchange–correlation functional. The numerical integration grid was set to fine accuracy. The global orbital cutoff radius was set to 5.0 Å. The Self-consistent field SCF convergence threshold was set to 1×10^-6^ Ha, and the geometry optimization convergence criteria were 1×10^-5^ Ha for energy and 2×10^-3^ Ha Å^-1^ for maximum force. Frontier molecular orbital energies (HOMO, LUMO) and the corresponding electron-density isosurfaces were extracted from the converged wavefunctions, and the HOMO-LUMO gaps (ΔE) were obtained from the Kohn-Sham eigenvalues. All orbital visualizations were generated using the built-in DMol^3^ visualization tools. The ESP was mapped onto the molecular van der Waals surface, yielding the color-coded maps presented in this study within the range of -0.039 to 0.039 (a.u.).

The following formula was used to calculate the binding energy values:

$E_{binding}$ =$E_{{Li}^{+} + COF}-E_{{Li}^{+}}-E_{COF}$

where $E_{{Li}^{+} + COF}$, $E_{{Li}^{+}}$, and $E_{COF}$ represent the energies of Li^+^+COF, original Li^+^, and original COF, respectively.


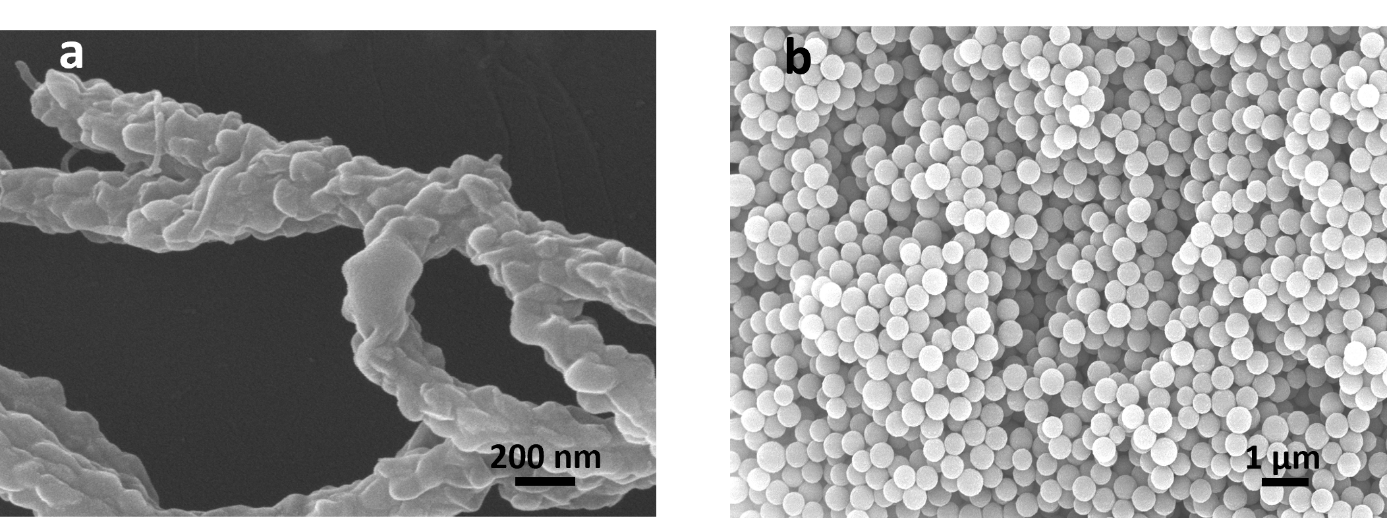


**Figure S1.** (a) SEM image of F-COF@CNT nanocables (b) SEM image of F-COF spheres.


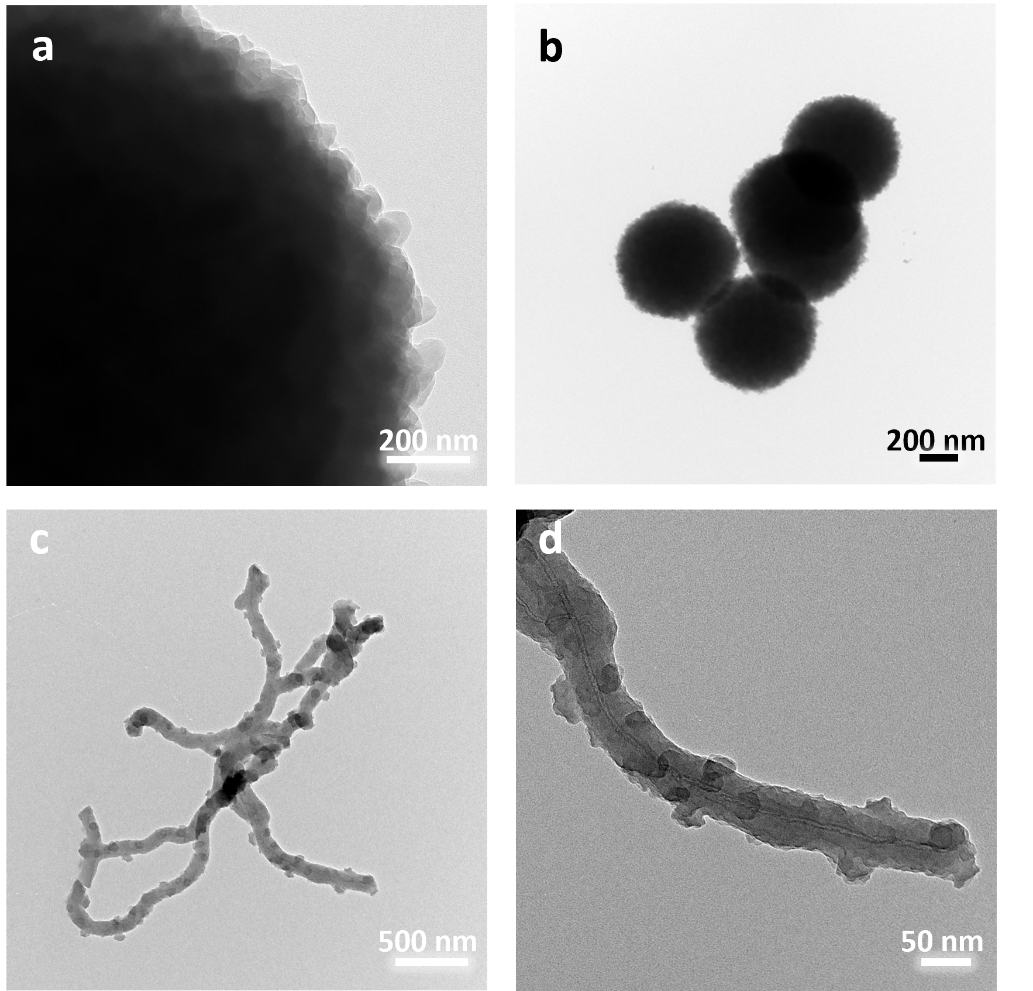


**Figure S2.** (a-b) TEM images of F-COF spheres, (c-d) TEM images of F-COF@CNT nanocables.


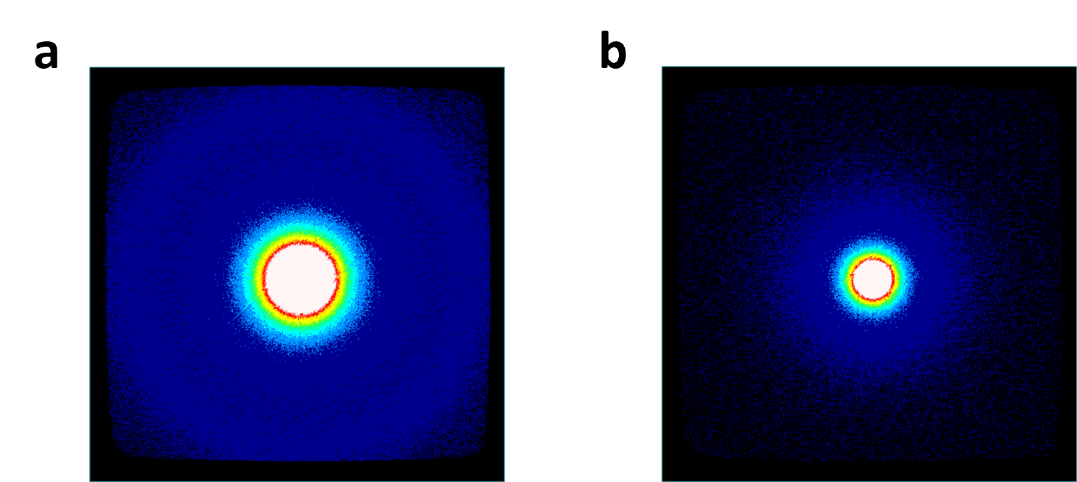


**Figure S3.** (a) SAXS pattern of F-COF and (b) F-COF@CNT.


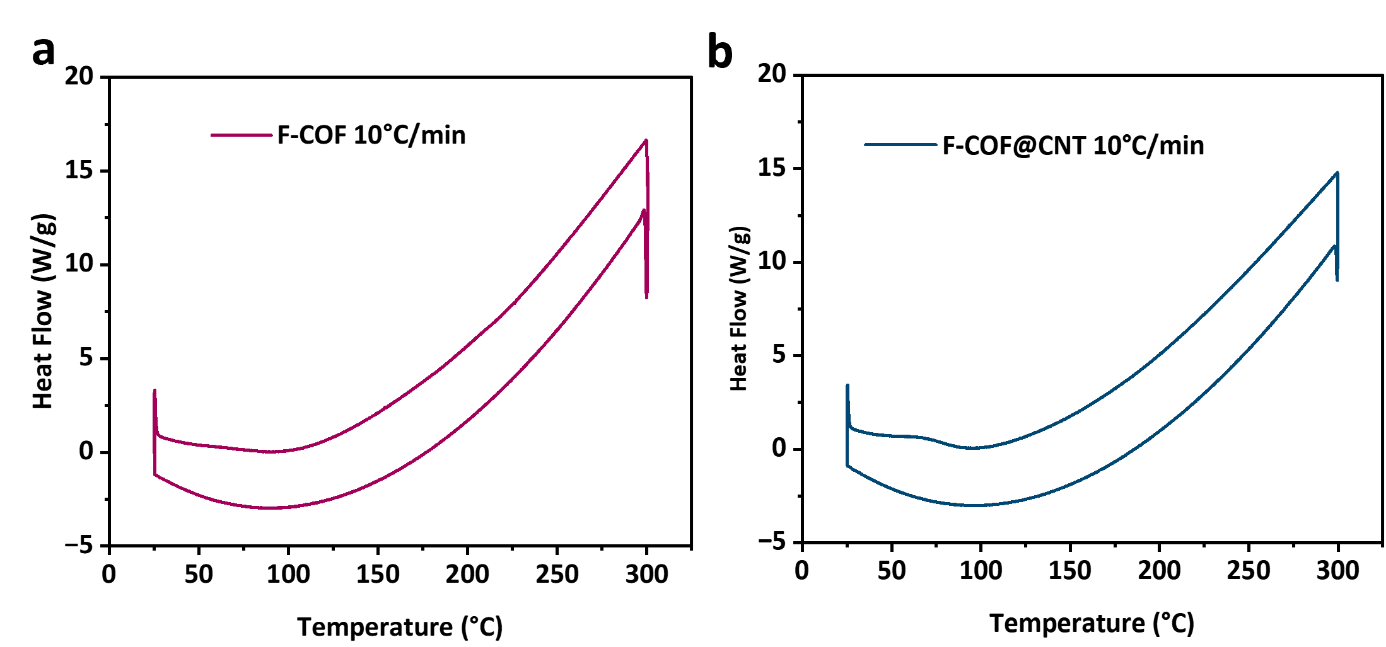


**Figure S4.** DSC curves of (a) F-COF and (b) F-COF@CNT recorded at a heating rate of 10 ℃ min^-1^.


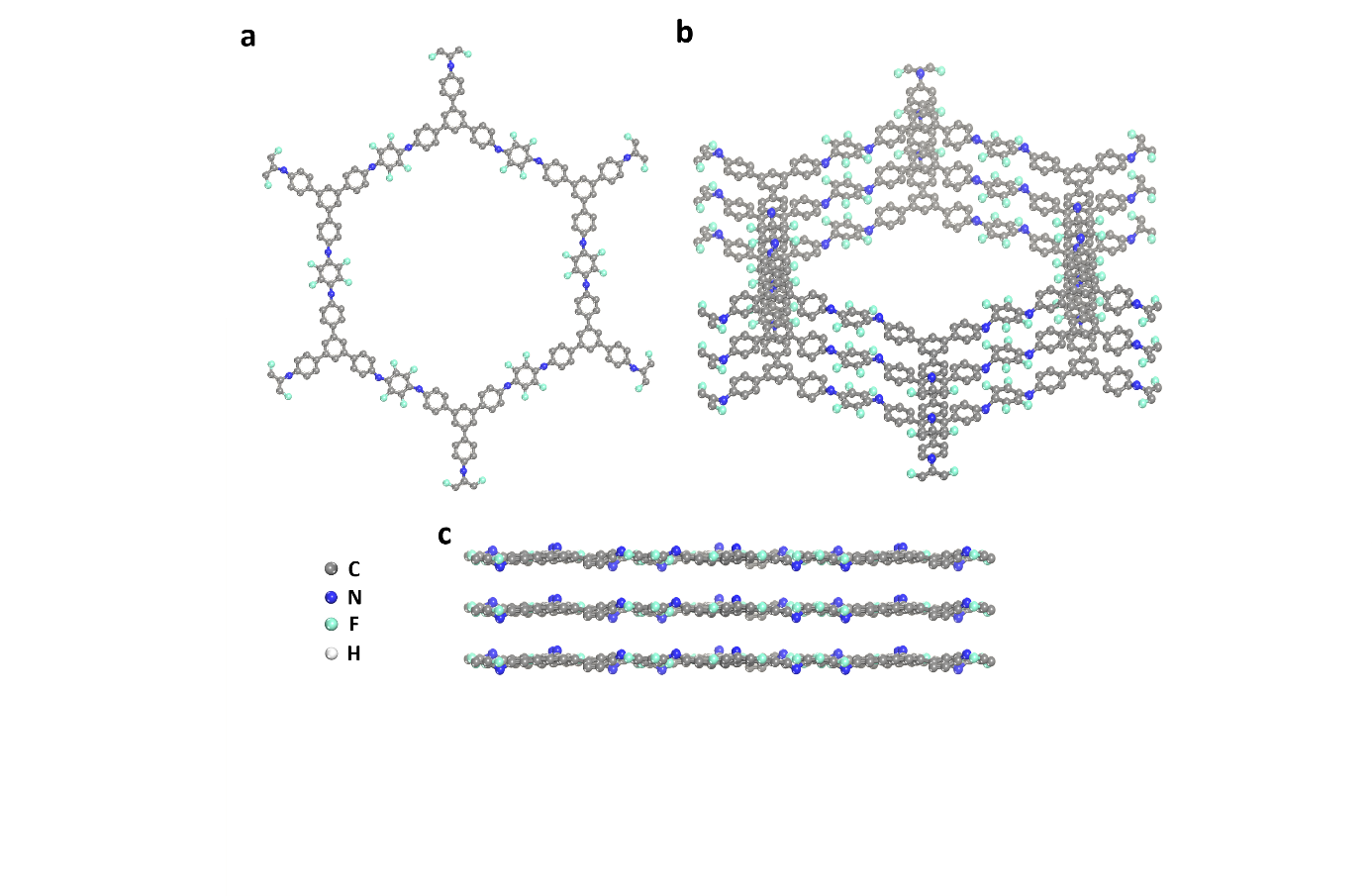


**Figure S5.** Structural models of the fluorinated covalent organic framework (F-COF). (a) Top view of the single-layer F-COF showing the hexagonal pore architecture. (b) Perspective view illustrating the extended framework connectivity and pore channels. (c) Side view of the multilayer F-COF, highlighting the ordered π–π stacking along the out-of-plane direction.


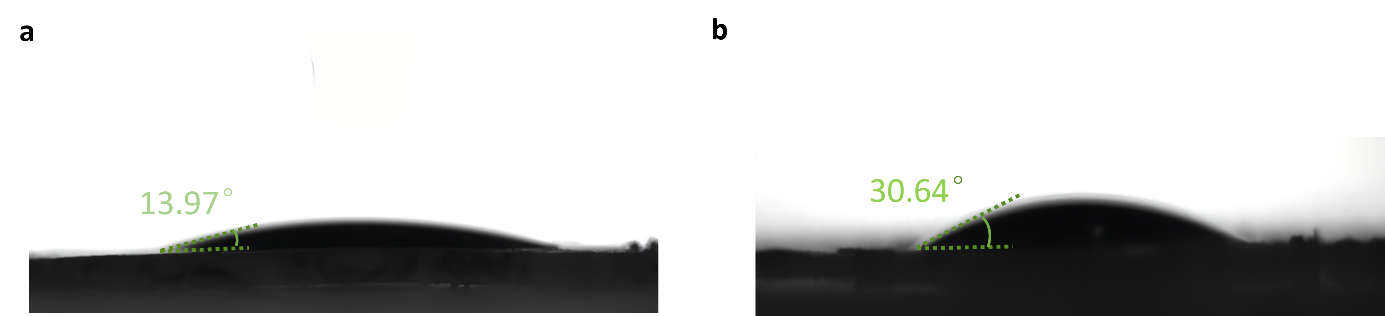


**Figure S6.** (a) Contact angle test of F-COF@CNT and (b) F-COF with the electrolyte 1 M LiPF_6_ in DMC/EC (1:1 in volume).


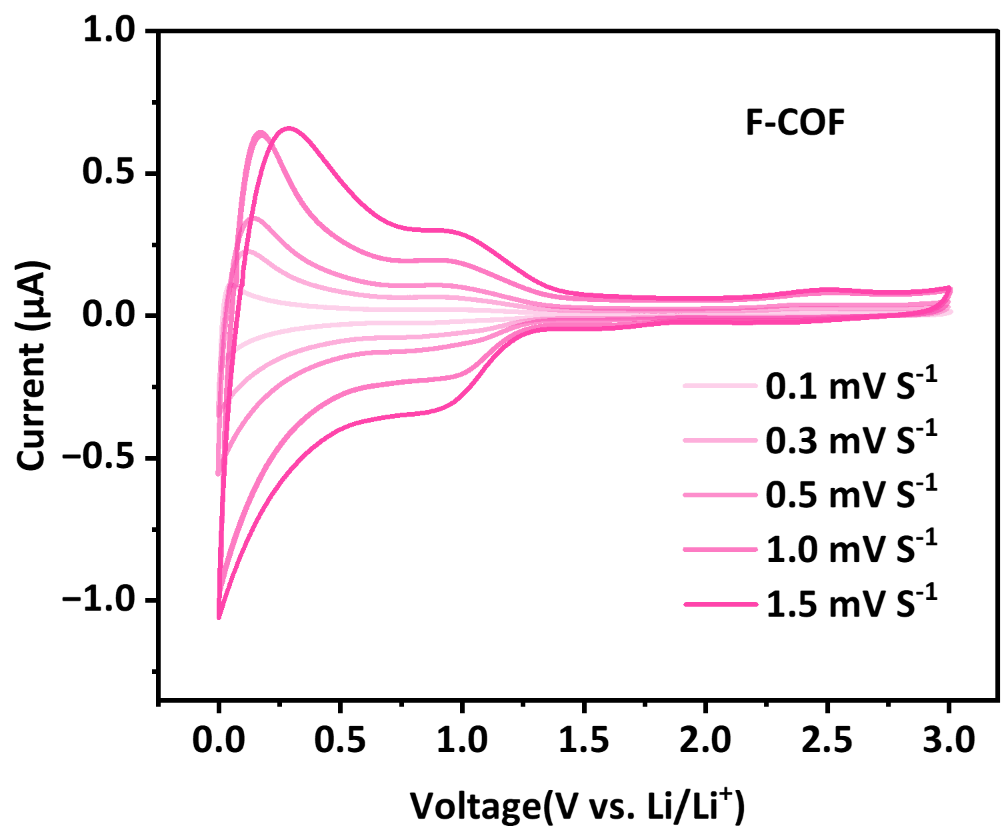


**Figure S7.** CV curves of F-COF from 0.1 to 1.5 mV s^-1^.


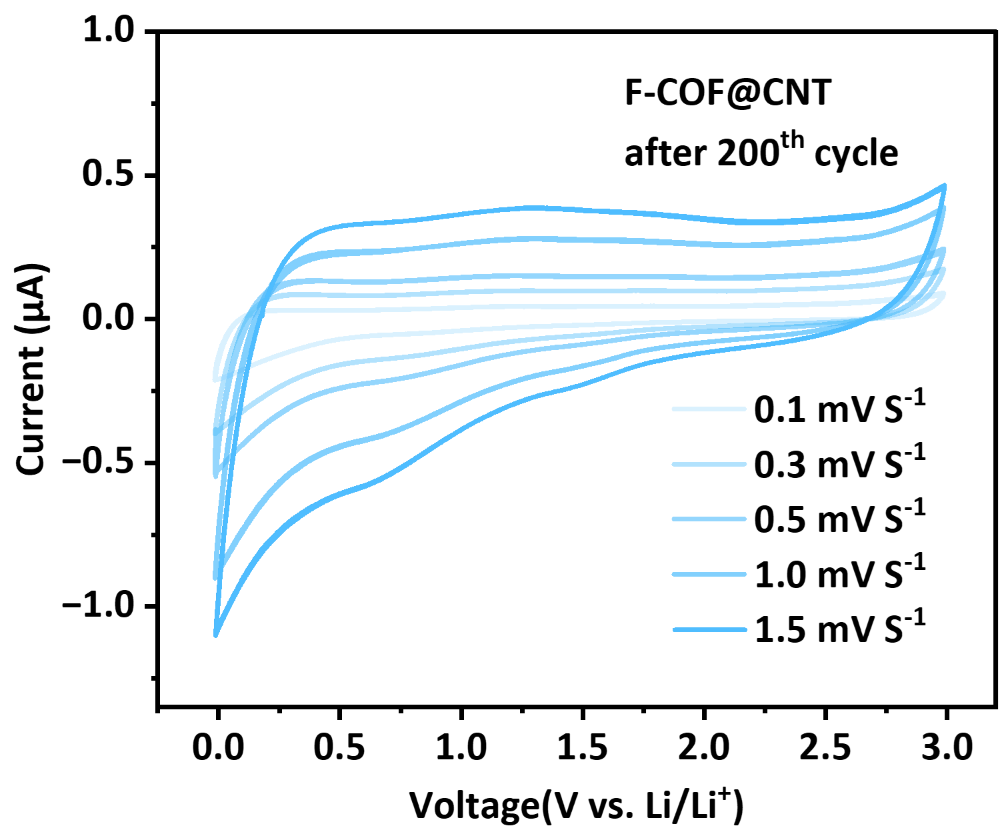


**Figure S8.** CV curves of F-COF@CNT after 200 cycles from 0.1 to 1.5 mV s^-1^.


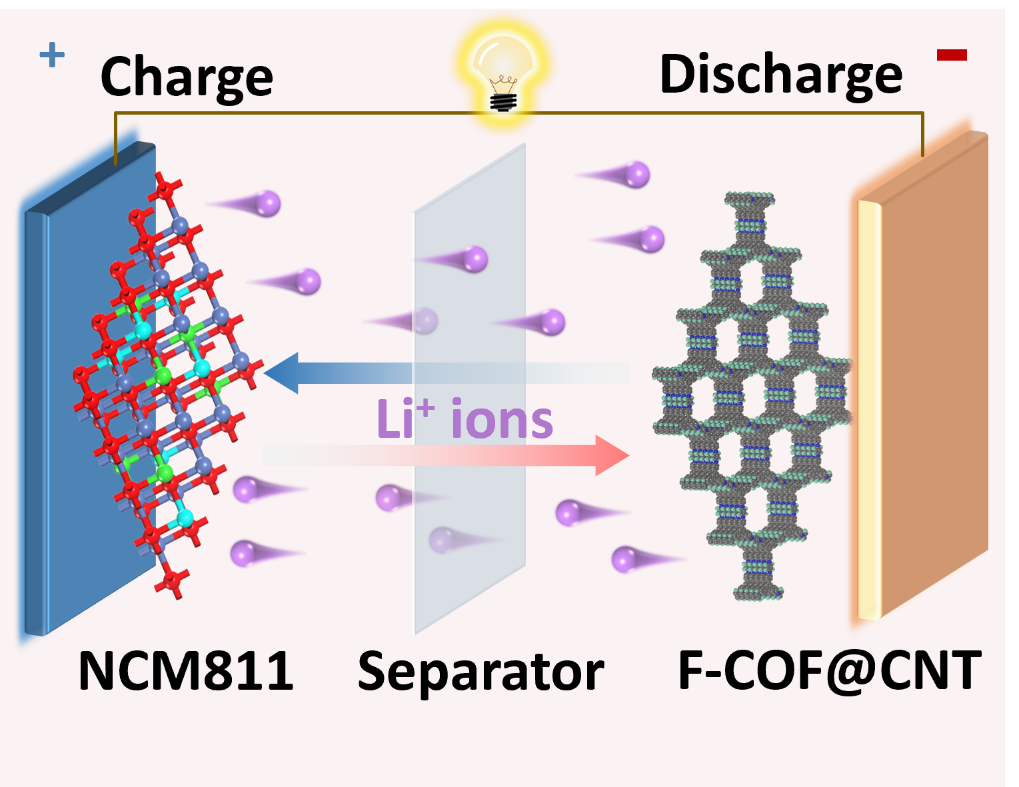


**Figure S9.** Schematic representation of the working mechanism of the NCM811||F-COF@CNT nanocables full battery.


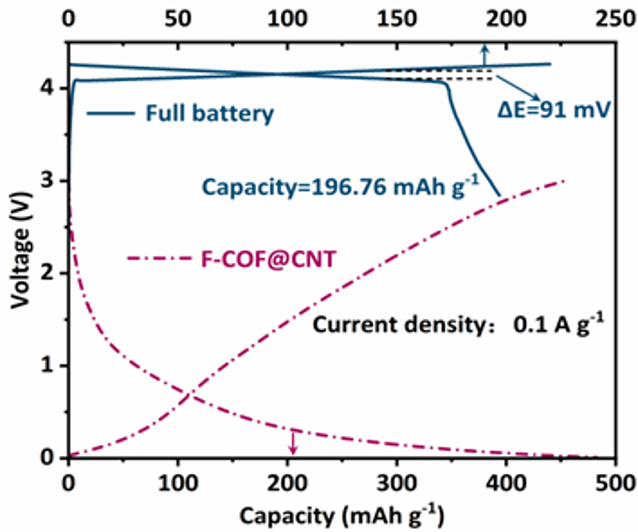


**Figure S10.** F-COF@CNT nanocables and NCM811||F-COF@CNT nanocables full battery typical charge/discharge profiles.





**Figure S11.** Prolonged cycling performance of NCM811||F-COF@CNT nanocables full battery

**

**

**Figure S12.** Cycle performance of pure CNTs.

**Table 1.** Comparison of different anode materials for lithium-ion batteries.

| **Materials** | **Max-specific capacity(mAh g^-1^)** | **Current density(A g^−1^)** | **Cycle number** | **Reference** |
| --- | --- | --- | --- | --- |
| MNO  Graphite  DCB-COF  TP-AZO-COF  TThpp  PDASA  TpBpy  LVO/C  Spruce Hard Carbon  Li_4_Ti_5_O_12_  PA-COF  F-COF@CNT | 179  315  452/387  ~400  384  120  370  169  ~156  155.6  401  466.04 | 0.1  7.4  0.2/0.4  1  1  1  1  ~10  ~0.2  ~1.75  1  0.1 | 720  1000  200/500  3000  200  1000  1000  500  150  500  1000  200 | 41  42  43  44  45  46  47  48  49  50  51  This work |
